# Evolutionary and Integrative Analysis of Gibberellin-Dioxygenase Gene Family and Their Expression Profile in Three Rosaceae Genomes (*F. vesca*, *P. mume*, and *P. avium*) Under Phytohormone Stress

**DOI:** 10.3389/fpls.2022.942969

**Published:** 2022-07-07

**Authors:** Irfan Ali Sabir, Muhammad Aamir Manzoor, Iftikhar Hussain Shah, Farhat Abbas, Xunju Liu, Sajid Fiaz, Adnan Noor Shah, Songtao Jiu, Jiyuan Wang, Muhammad Abdullah, Caixi Zhang

**Affiliations:** ^1^School of Agriculture and Biology, Shanghai Jiao Tong University, Shanghai, China; ^2^School of Life Sciences, Anhui Agricultural University, Hefei, China; ^3^College of Horticulture, South China Agricultural University, Guangzhou, China; ^4^Department of Plant Breeding and Genetics, The University of Haripur, Haripur, Pakistan; ^5^Department of Agricultural Engineering, Khwaja Fareed University of Engineering and Information Technology, Rahim Yar Khan, Pakistan

**Keywords:** *PavGAox*, characterization, gene duplication, subcellular localization, qRT-PCR

## Abstract

The gibberellin-dioxygenase (GAox) gene family plays a crucial role in regulating plant growth and development. GAoxs, which are encoded by many gene subfamilies, are extremely critical in regulating bioactive GA levels by catalyzing the subsequent stages in the biosynthesis process. Moreover, GAoxs are important enzymes in the GA synthesis pathway, and the GAox gene family has not yet been identified in Rosaceae species (*Prunus avium* L., *F. vesca*, and *P. mume*), especially in response to gibberellin and PCa (prohexadione calcium; reduce biologically active GAs). In the current investigation, 399 GAox members were identified in sweet cherry, Japanese apricot, and strawberry. Moreover, they were further classified into six (A-F) subgroups based on phylogeny. According to motif analysis and gene structure, the majority of the *PavGAox* genes have a remarkably well-maintained exon–intron and motif arrangement within the same subgroup, which may lead to functional divergence. In the systematic investigation, *PavGAox* genes have several duplication events, but segmental duplication occurs frequently. A calculative analysis of orthologous gene pairs in *Prunus avium* L., *F. vesca*, and *P. mume* revealed that GAox genes are subjected to purifying selection during the evolutionary process, resulting in functional divergence. The analysis of *cis*-regulatory elements in the upstream region of the 140 *PavGAox* members suggests a possible relationship between genes and specific functions of hormone response-related elements. Moreover, the *PavGAox* genes display a variety of tissue expression patterns in diverse tissues, with most of the *PavGAox* genes displaying tissue-specific expression patterns. Furthermore, most of the *PavGAox* genes express significant expression in buds under phytohormonal stresses. Phytohormones stress analysis demonstrated that some of *PavGAox* genes are responsible for maintaining the GA level in plant-like *Pav co4017001.1 g010.1.br*, *Pav sc0000024.1 g340.1.br*, and *Pav sc0000024.1 g270.1.mk*. The subcellular localization of *PavGAox* protein utilizing a tobacco transient transformation system into the tobacco epidermal cells predicted that GFP signals were mostly found in the cytoplasm. These findings will contribute to a better understanding of the GAox gene family’s interaction with prohexadione calcium and GA, as well as provide a strong framework for future functional characterization of GAox genes in sweet cherry.

## Introduction

Gibberellins (GAs), a class of diterpenoid phytohormones first discovered by Dr. E. Kurosawa in 1926, regulate a variety of processes in plants, including stem elongation, anther growth, dormancy regulation, and flower initiation (Vera-Sirera et al., 2016; Pan et al., 2017). Several studies proved that dwarfism has been linked to deficiencies in gibberellin (GA) levels or signaling (Fukazawa et al., 2017). In agriculture, GA levels are extensively regulated to promote seedless grapefruit development; slow fruit senescence in lemons and oranges; improve fruit setting in pears, apples, and mandarins; enhance stem elongation in sugarcane; and reduce growth in apple, canola, and cotton (Hedden and Phillips, 2000). GAs also regulate many crucial physiological processes like fruit senescence (Serrani et al., 2007), shoot elongation (Junttila et al., 1992), seed germination (Tyler et al., 2004; Ayele et al., 2006), and leaf expansion (Hedden and Proebsting, 1999).

There have been up to 136 distinct gibberellin molecules identified so far, and the majority of them had been classified as catabolites or biosynthetic intermediates of bioactive Gas, including GA_1_, GA_3_, GA_4_, and GA_7_ (Hedden and Thomas, 2012). GA biosynthesis comprises three reaction stages (Olszewski et al., 2002; Li et al., 2017). In the first step, GA production starts from the conversion of geranylgeranyl diphosphate (geranylgeranyl-PP) into metabolite ent-Kaur-16-ene in the presence of ent-kaurene synthase (KS) and ent-copalyl diphosphate synthase (). Afterward, ent-kaur-16-ene is converted into GA12 and GA53 through P450-dependent monooxygenase cytochromes, which are ent-kaurene oxidase (KO) and ent-kaurenoic acid oxidase (KAO), respectively. Following three continuous processes, the formation of numerous GAs occurs in the final stage of biosynthesis through two pathways: early-13-hydroxylation and non-13-hydroxylation. GA 20-oxidases (GA20ox) and GA 3-oxidases (GA3ox), which belong to the 2-oxoglutarate-dependent dioxygenase (2-ODD) family, are vital enzymes in a process of oxidation steps that transform GA12 and GA53 into different GA intermediates and bioactive GAs (GA1 and GA4). GA 2-oxidases (GA2oxs) are special enzymes that belong to the 2-ODD family and are involved in GA degradation. The bioactive GAs (GA1 and GA4), as well as their immediate precursors (GA20 and GA9), are suppressed by these enzymes. Moreover, investigations have demonstrated that all GA2ox, GA3ox, and GA20ox sequences are related to the 2-ODD superfamily, which has a significant level of homology along with the functional domains (Kaul et al., 2000; Yamaguchi, 2006). Moreover, prohexadione calcium (PCa) is synthesized by 2-oxoglutarate, which is a key dioxygenase co-substrate. It has been discovered that it inhibits 3b-hydroxylation, which mediates the late stages of GA synthesis (Ilias and Rajapakse, 2005; Kim et al., 2007). PCa reduced the mobility of biologically active GAs while augmenting the levels of inactive GA20 (Rademacher, 2000). Additionally, PCa effectively regulates the GA level in plants, which also plays a vital role in regulating dormancy. Previous research has also demonstrated the importance of (GA, ABA) metabolic and signaling properties of numerous plant hormones, as well as their putative relationship in the retention and breaking of dormancy (Shu et al., 2016). Bud dormancy is a physiological stage of woody deciduous plants that allows them to endure for longer periods under unfavorable environments. It is revealed by stopping growth, pausing cell division, reduction in respiratory activity, and metabolic procedures (Faust et al., 1997). Dormancy plays a critical role in optimum blooming and fruit set (Fadón and Rodrigo, 2018). Furthermore, the previous investigation provides evidence on the role of GAs in the triggering of growth halting, which results in the reduction in active GA levels in dormant plants (Cooke et al., 2012). Numerous studies have reported high levels of endogenous GA or a higher expression of GA biosynthetic genes during the perennial end dormant bud formation or the natural bud endodormancy cycle. The endogenous ABA/GA_3_ ratio in *Prunus avium* L. flower buds increased throughout natural dormancy induction and reduced when dormancy was released (Chengguo et al., 2004). Peach-dormant buds were found to have higher levels of GA in chilling hours (Frisby and Seeley, 1993). Previous studies illustrated that when aspen-dormant buds were treated with low temperature, the expression pattern in GA3ox and GA20ox gene families was higher, while lower expression in the GA2ox family was identified (Karlberg et al., 2010; Rinne et al., 2011). Studies demonstrated that GA_3_ improved the dormancy release in Elberta peach buds, while the same was also observed in Japanese apricot flower buds when treated with GA4 (Donoho and Walker, 1957; Zhuang et al., 2013).

Sweet cherry is an extremely temperature-sensitive perennial plant (Maurya et al., 2018; Kose and Kaya, 2022). Dormancy is strongly influenced by external temperatures, and variations in the timing of bud break and flowering have been associated with global warming (Kaya et al., 2021; Tominaga et al., 2022). Bud break and flowering dates for sweet cherry in the northern hemisphere were extended in the spring, raising the chances of late frost damage (Vitasse et al., 2014; Bigler and Bugmann, 2018; Kaya and Kose, 2022a,b), whereas inadequate cold accumulation during winter could contribute to inadequate dormancy release and limited bud break rate (Erez, 2000; Atkinson et al., 2013). These phonological differences have a direct influence on fruit crop output, which might result in significant economic losses (Snyder and Melo Abreu, 2005). Current findings revealed that gibberellin plays an important role in sweet cherry dormancy regulation. Furthermore, identifying the GA metabolic regulatory system in *P. avium* might provide a molecular framework for a targeted breeding program, as well as provide a basic framework for understanding the role of dormancy. Gibberellin oxidase is a crucial synthase and catalyst which is involved in the interconversion of various GAs in the gibberellin biosynthesis pathway’s final step. However, no reports on *P. avium* seem to be published. We identified GAox genes in the *P. avium* genome database. For the *PavGAox* genes, fundamental physicochemical properties, gene structure, conserved domains, and selected evolutionary relationships were extensively examined. RNA sequencing (RNA-seq) and quantitative real-time PCR (qRT-PCR) were used to examine *PavGAox* gene expression patterns in different organs and in response to various phytohormones. Furthermore, the gibberellin dioxygenase gene family in sweet cherry has received relatively little systematic and detailed research. As a result, the purpose of this study was to investigate the characteristics of the GAox gene family and identify the key genes in sweet cherry that respond to GA to regulate biological functions, as well as the dormancy process, which has been negatively impacted by global rwarming.

## Materials and Methods

### Collection, Identification, and Molecular Characteristics of GAox Genes

Rosaceae genome sweet cherry (*P. avium*, v1.0.a1) and Japanese apricot (*P. mume*, v1.0) sequences were downloaded from the Genome Database for Rosaceae (GDR)^1^ (Zhang et al., 2012; Shirasawa et al., 2017), while the Joint Genome Institute (JGI) Data Portal^2^ was used to extract the genome of strawberry (*Fragaria vesca*, v4.0) (Jung et al., 2014). The protein sequence alignment was obtained in a specific Stockholm format with the GAox domain (Pfam; PF03171 and PF14226), and Hmmbuild was used to develop a model from the alignment. The Hmmsearch program was utilized to explore the genome database of three Rosaceae species (*F. vesca*, *P. mume*, and *P. avium*) for all potential GAox genes. Second, BioEdit software was utilized to retrieve 399 potential GAox protein sequences from the three Rosaceae genomes through 16 protein sequences of Arabidopsis as queries in BlastP (E value cut-off of 1 × 10^–5^). The sequences of all GAox proteins were aligned, and repetitive GAox genes were eliminated. Moreover, InterProScan^3^ (Finn et al., 2006), SMART^4^ (Letunic et al., 2012), and Pfam databases^5^ (Finn et al., 2006) were used to verify all elected GAox genes. Subsequently, ExPASY web^6^ was utilized to estimate physicochemical properties (isoelectric point, amino acid length, and weight) (Abdullah et al., 2018a), while CELLOGO tool software^7^ was used to assess subcellular localization (Manzoor et al., 2020, 2021a).

### Phylogeny and Alignment Analysis of the GAox Gene Family

ClustalX software was used to align the 399 GAox full-length protein sequences obtained from the three Rosaceae species (*P. avium*, *P. mume*, and *F. vesca*), along with 16 GAox full-length protein sequences of *Arabidopsis thaliana* while utilizing default parameters (1,000 bootstrap, pairwise deletion) (Thonpson, 1997). Molecular Evolutionary Analysis (MEGA-X) was utilized to construct the phylogenetic tree through the maximum likelihood method (ML-M) (Tamura et al., 2011). Eventually, itol online program^8^ was used to illustrate the phylogenetic trees (Letunic and Bork, 2019).

### Conserved Motif and Exon–Intron Structural Analysis

GSDS (Gene Structure and Display server v.2.0)^9^ was utilized to visualize the vital gene structure characteristics like conserved elements, composition, and position intron–exon (Hu et al., 2015; Abdullah et al., 2018b; Manzoor et al., 2021a; Sabir et al., 2022). MEME (the Motif Elicitation)^10^ program was used to illustrate the conserved motifs (Bailey et al., 2015).

### Chromosomal Distribution and Conserved Domain Analysis

All GAox gene chromosomal positions were retrieved from a genome database (see text footnote 1), and Mapinspect software^11^ was used for the visualization of these positions (Manzoor et al., 2021b,c). Moreover, the Conserved Domain Architecture Retrieval Tool (CDART), HMMER program^12^, and protein family database (Pfam)^13^ were utilized for obtaining the conserved domain of GAox protein of *P. avium*, *Fragaria vesca*, and *P. mume* (Johnson et al., 2010; Mistry et al., 2021).

### *Cis*-Element Analysis

All sweet cherry GAox genes and promoter sequences were found from the start codon along with 2,000-bp upstream sequences, and PlantCARE database online webtools^14^ were utilized to anticipate and filter all *cis*-elements (Lescot et al., 2002).

### Gene Duplications, Collinearity Relationships, and ka/ks Analysis

Collinearity assessment was conducted using MCScanX (Multiple Collinearity Scan toolkit) through BLASTP (E < 1e^–5^) across three Rosaceae genomes (Wang et al., 2012). The Multiple Collinearity Scan toolkit was used to identify several types of duplications like whole-genome duplication (WGD), dispersed duplication (DD), proximal duplication (PD), transposed duplication (TRD), and tandem duplication (TD) in *P. avium*, *P. mume*, and *F. vesca*. Circos and TBtools were used to identify collinearity relations and gene duplications. The Plant Genome Duplication Database^15^ was used to acquire synonymous mutation rates (ks) and the non-synonymous (ka) rates for relevant duplication gene pairs (Cao et al., 2013; Abdullah et al., 2018a; Manzoor et al., 2021b). MAFFT software and calculators^16^ were utilized to calculate the ka/ks ratio of each duplicated gene pair, as well as numerous alignments (Qiao et al., 2019).

### Plant Materials

“Royal Lee” (sweet cherry cultivar) was cultivated at Shanghai Jiao Tong University experimental farm area in Minhang district, Shanghai, China (31.25°N, 121.48°E). Gisela 6 (G6) was utilized as rootstock for grafting diploid cultivars. All trees were planted at 5–6 m spacing, while the same agricultural practices were used on all trees. A measure of 200 μl prohexadione calcium (PCa) and 500 μl gibberellin (GA_4+7_) were sprayed on fully mature buds. All bud samples and the control were collected on the first, third, and sixth days. Furthermore, all experimental materials were freeze-dried and kept at −80°C until use.

### Subcellular Localization

For analyzing the subcellular localization in Tobacco (*Nicotiana tabacum*) epidermal cells, the coding sequence of *PavGAox* gene with codon was inserted into the 35Spro-eGFP vector with gene-specific primers to generate p35S:: *Pav_sc0000465.1_g550.1.mk*::eGFP construct. The recombinant constructed plasmid was transferred into *Agrobacterium tumefaciens* strain EHA105 by electroporation. A positive colony was cultured in LB medium containing antibiotics at 28 °C in the dark. After centrifugation, the pellet was collected and resuspended in infiltration buffer (Wani et al., 2020). Afterward, transient expression was performed through the agro-infiltration method. The green fluorescence protein in tobacco leaves was monitored at 4–6 days post-agro-infiltration and visualized by using a Leica SP8 confocal microscope excitation, 488 nm, emission 500–550 nm.

### Transcriptional Analysis of Sweet Cherry GAox Genes

RNA-seq data were carried out with accession numbers SRR8984402, SRR8984360, SRR8984367, SRR8984382, SRR8984344, SRR8984381, SRR8984359, SRR8984403, SRR8984342, and SRR8984366 of *P. avium* at various dormancy phases (organogenesis, paradormancy, endodormancy, ecodormancy). The SRA toolkit was used to encrypt the data from the SRA database into the FASTQ version. Hisat2 software was utilized to align each dataset to the reference genome using default settings. Using the StringTie software, the expression level was calculated in transcripts per kilobase million (TPM). Finally, TBtools was used to visualize the heat map (Chen et al., 2018).

### RNA Extraction, Reverse Transcription, and qRT-PCR

To further investigate the functional role of GAox genes in flowers, buds, and fruits, we performed real-time quantitative PCR analysis to examine the expression patterns of selected genes. The total RNA of GA treatment and PCa treatment was isolated by using the RNAprep Pure Plant Kit (Tiangen). First-strand cDNA synthesis was reversed with gDNase Eraser-treated RNA (1 μg) by Prime Script RT reagents Package along with Takara. The anti-sense and sense primers were designed by GeneScript online software^17^. The primer sequences used in this study are shown in Supplementary Table 1. Three technical and biological duplicates per sample were used in the qRT-PCR experiments. qRT-PCR was performed with a cDNA template (2 μl) along with reverse and forward primers (0.8 Lμl). A measure of 10 μl SYBR premix ExTaq II was used, and nuclease-free water was added to a final volume of 20 μl. Temperature was set as follows: 50°C for 2 min, 95°C for 30 s, accompanied by 40 cycles of 95°C for 15 s, 60°C for 20 s, and 72°C for 20 s 4°C for sweet cherry. We used actin protein as an internal reference and applied the 2?-^ΔΔ]Ct^ method to calculate the relative transcription level of target genes (Livak and Schmittgen, 2001; Letunic et al., 2012).

## Results

### Identification and Physico-Chemical Properties of GAox Genes in Rosaceae Species

**FIGURE 1:**
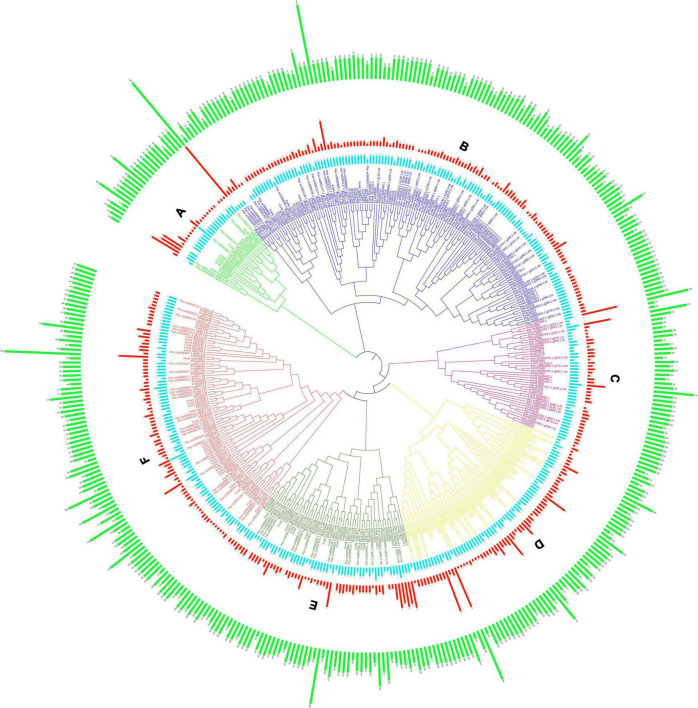
Phylogenetic tree of GAox protein of *P. avium*, *P. mume*, *F. vesca*, and *A. thaliana*. Each color representing a subfamily **(A–F)** of GAox genes. Green, red, and light blue bars indicate length of amino acids, intron, and domain numbers, respectively. The phylogenetic tree was constructed with the itol software.

All GAox genes from *P. avium*, *P. mume*, and *F. vesca* genomes were identified using the *A. thaliana* GAox sequence as a query file. The GAox genes were identified in the Rosaceae (*P. avium*, *F. vesca*, and *P. mume*) genome database using two methods, namely, local BLASTP analysis and HMM search (Supplementary Table 2). Finally, 399 Gaox genes were discovered and utilized for future research. There were 140 sweet cherry, 146 Japanese apricot, and 113 strawberry *PavGAox* genes. The evolutionary relationship between *P. mume*, *F. vesca*, and *P. avium* was investigated. Subsequently, the full-length protein sequences of *P. avium* (140), *P. mume* (146), *F. vesca* (113), and *A. thaliana* (16) were aligned using clustalX, and a phylogenetic tree was built through Molecular Evolutionary Genetics Analysis (MEGA-X) software. Furthermore, we utilized the maximum likelihood method (ML-M) for phylogeny analysis along with 1,000 times with bootstrapping values with other default parameters (Figure 1). All GAox-genes discovered in the three Rosacea species were divided into six subfamilies (A–F). The maximum GAox members (71) were found in subfamily B, while the lowest GAox members (34) were found in subfamily A (Figure 1).

Moreover, in strawberry, GAox protein contained 153–1,442 amino acids, with an average of 391.12. In sweet cherry and Japanese apricot, the length of amino acid sequences ranged from 101 to 745 and from 69 to 1,451, with an average of 298.12 and 333.76, respectively. The molecular weight of *PavGAox* members ranged between 11,302 and 82,709 kDa, with an average of 33,654.55 kDa, while in strawberry and Japanese apricot, the molecular weight ranged from 17,321 to 166,637.2 kDa and from 7,679.97 to 163,783.1 kDa, with an average of 44,200.34 and 37,670.11 kDa, respectively. The values of the isoelectric point (pI) in *P. avium* varied from 4.68 to 9.86, with an average of 6.15, while pI in *P. mume* and *F. vesca* varied from 4.52 to 10 and 4.59 to 8.96, with an average of 5.91 and 5.71, respectively (Supplementary Table 2).

### Motif Analysis and Gene Structure Analysis

**FIGURE 2:**
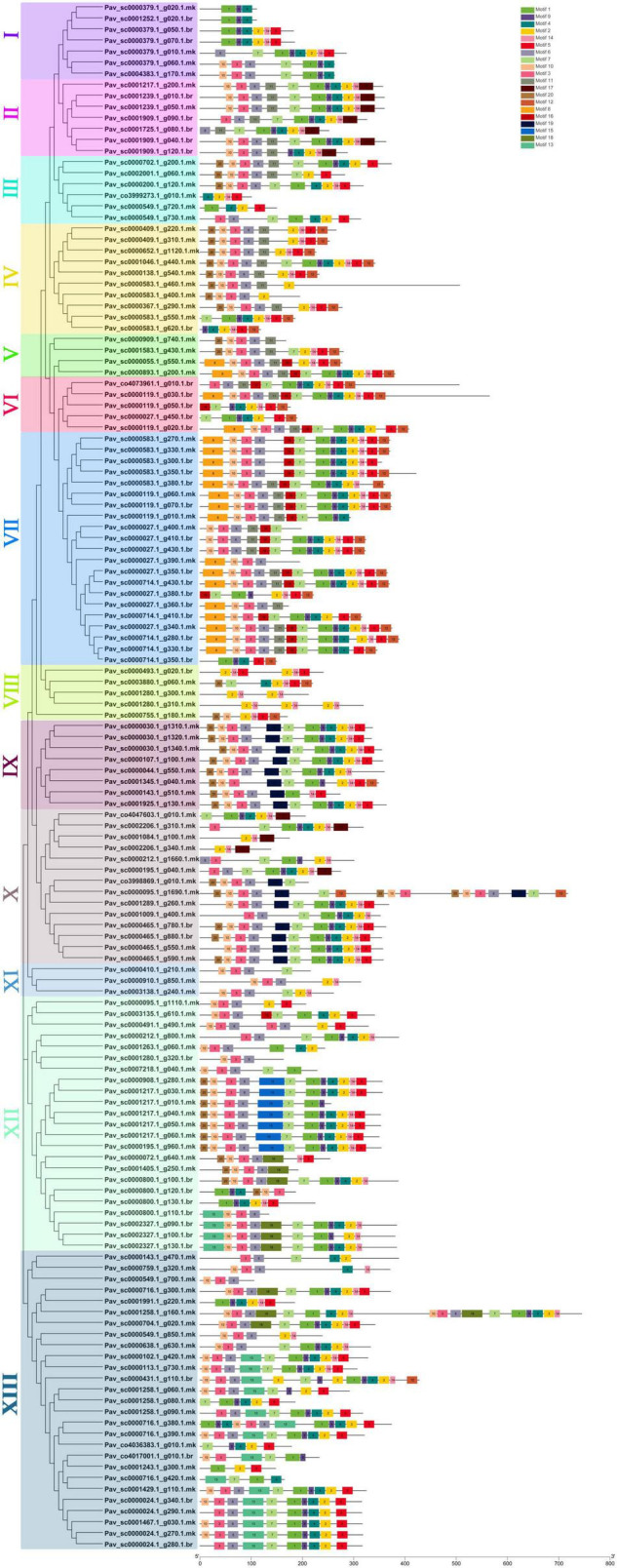
Phylogenic relationships and conserved protein motif composition for GAox family proteins in sweet cherry. The motif composition of PavGaox protein (1–20) is indicated by different colored boxes with specific motif numbers, and figure legends are mentioned on the top.

**FIGURE 3:**
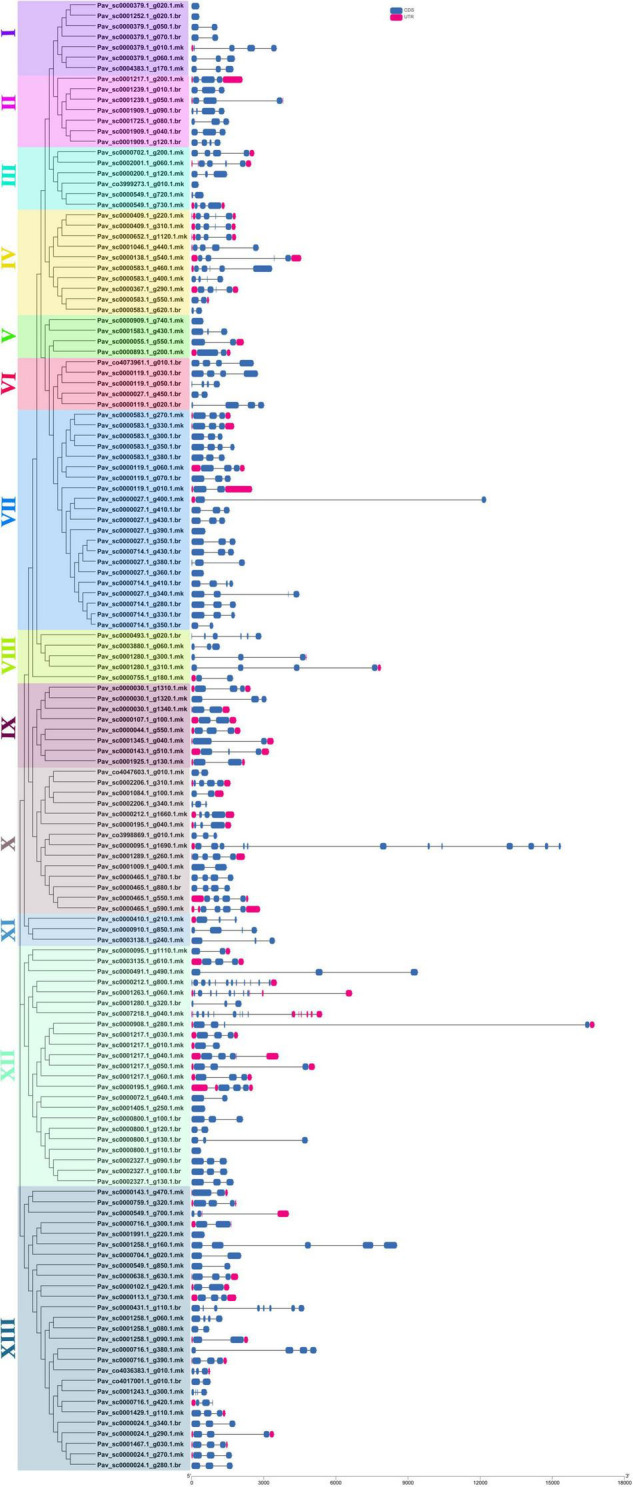
Phylogenic relationships and gene structure for GAox family proteins in sweet cherry. The relative position and size of the exon can be estimated using the scale at the bottom. Blue boxes, black lines, and red boxes represent exons, introns, and UTR, respectively.

Totally, 20 motifs were identified from 140 *PavGAox* genes, and at least three motifs were recognized in all *PavGAox* genes (Figure 2). The maximum number of motifs (19) was discovered in *Pav_sc0001258.1_g160.1.mk* (subfamily-XIII), while 17 motifs were found in *Pav sc0000095.1 g1690.1.mk* (subfamily-X). Meanwhile, *Pav sc0000549.1 g700.1.mk* (subfamily-XIII), *Pav sc0001280.1 g320.1.br* (subfamily-XII), *Pav sc0002206.1 g340.1.mk*, *Pav sc0001084.1 g100.1.mk* (subfamily-X), *Pav sc0000379.1 g020.1.mk*, and *Pav sc0001252.1 g020.1.br* (subfamily-I) had the least number (3) of motifs. Different subgroups had some specific motifs, for example, motif 17 was only detected in subgroups II and X, representing that the proteins in all these subgroups may be subsidizing for some crucial functions (Figure 2). Some motifs like 2, 4, and 14 were found in all subgroups, indicating that the addition of these motifs (2, 4, and 14) to the subgroups may have occurred *via* evolutionary processes, and these motifs may have significant roles. Figure 2 demonstrates that members of each subgroup with a strong biological relationship have identical motif compositions like in subfamily II (*Pav sc0001217.1 g200.1.mk*, *Pav sc0001239.1 g010.1.br*, and *Pav sc0001239.1 g050.1.mk*), subfamily IX (*Pav sc0000030.1 g1310.1.mk*, *Pav sc0000030.1 g1320.1.mk*, *Pav sc0000030.1 g1340.1.mk*, and *Pav sc0000107.1 g100.1.mk*), and subfamily XII (*Pav sc0001217.1 g040.1.mk*, *Pav sc0001217.1 g050.1.mk*, *Pav sc0001217.1 g060.1.mk*, and *Pav sc0000195.1 g960.1.mk*), suggesting that GAox proteins functioned similarly. Each GAox gene was examined using intron–exon analysis to further the advanced projection into the fundamental and structural variability of the GAox family of genes (Supplementary Table 2). Currently, the results illustrated that sweet cherry has the most introns/exons, with a range of 1–15/1–12 (Figure 3). A maximum number of introns were found in *Pav_sc0007218.1_g040.1.mk* (subfamily XII), while a minimum number of the introns were found in *Pav_sc0001405.1_g250.1.mk* and *Pav_sc0000800.1_g110.1.br* (subfamily XII) and *Pav_sc0000027.1_g390.1.mk* and *Pav_sc0000027.1_g360.1.br* (subfamily VII). Most of the *PavGAox* genes that were clustered in the same subfamily (subfamily VI) had a similar number of introns and exons. All members contained a similar number of introns and exons (3/4), except one member (*Pav_sc0000027.1_g450.1.br*).

### Syntenic Analysis and Chromosomal Distribution of GAox Genes

**FIGURE 4:**
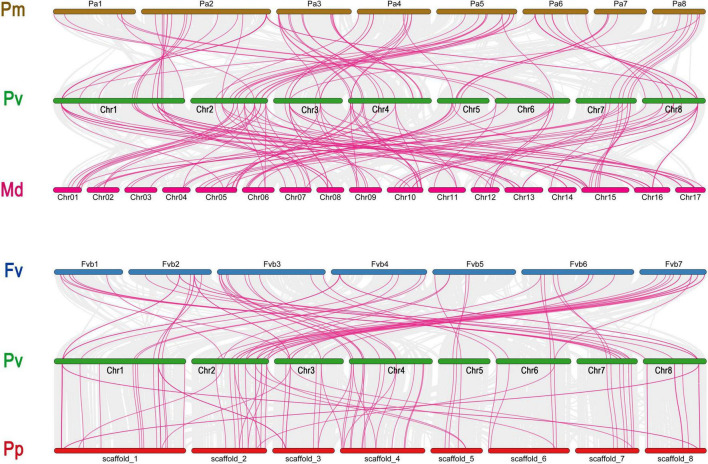
Collinearity relationship of GAox genes in *P. avium* and other Rosaceae species. Pa, Pp, Pm, and Fv indicate *P. avium*, *P. persica*, *P. mume*, and *F. vesca*, respectively.

There were 357 orthologous gene pairs identified across all Rosaceae genomes, which include 85 orthologous gene pairs between *Prunus avium* and *Prunus mume*, 119 orthologous gene pairs between *Prunus avium* and *Malus domestica*, 86 orthologous gene pairs between *Prunus avium* and *Prunus persica*, and 67 orthologous gene pairs between *Prunus avium* and *Fragaria vesca*, indicating a close relationship between these five Rosaceae genomes (Figure 4 and Supplementary Table 3).

The chromosomal distribution of GAox genes in sweet cherry was also examined. Overall, 118 *PavGAox* genes were found on eight chromosomes, and 22 genes were located on the scaffold. The highest number of *PavGAox* genes (35) was discovered on chromosome 1, while Chr8 had 25 *PavGAox* genes. Chr2, Chr6, and Chr7 contained 10, 6, and 7 GAox genes in the scattered formation, respectively, while Chr3, 4, and 5 had 8, 18, and 9 genes, which were distributed in cluster formation on chromosomes (Supplementary Figure 2).

### Mode of Gene Duplication Events and Ka/Ks Value

**FIGURE 5:**
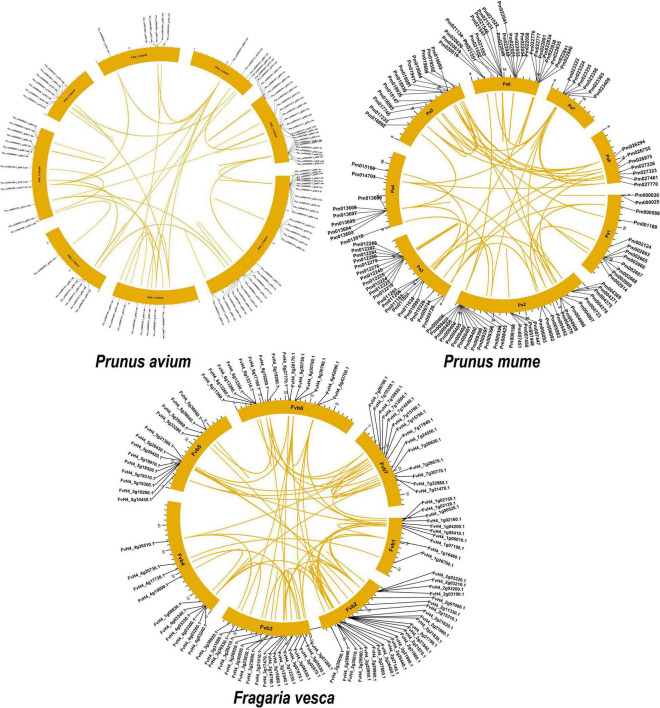
*Prunus avium*, *Prunus mume*, *Prunus persica*, and *Pyrus bretschneideri* have gene duplication and chromosomal localization. A colorful line links duplicated gene pairs.

We discovered numerous gene duplication events and their implications to GAox gene family expansion. Surprisingly, 243 duplicated gene pairs were found in three Rosacea species, with the highest number of duplicated gene pairs derived from dispersed duplications (75/243 genes pair), followed by tandem duplications (69/243 genes pair) and proximal duplications (40/243 genes pair), indicating that the GAox gene expression was predominantly associated with DSD, TD, and PD duplication events. Moreover, 34 transposed duplications (TRDS) and 25 whole-genome duplications (WGDs) were recognized in the GAox gene family (Figure 5). Furthermore, *P. avium* (26.5%), *P. mume* (29.59%), and *F. vesca* (35.80%) GAox genes had to participate in DSD, while 34.37, 28.57, and 23.45% GAox genes participated in tandem duplication, respectively.

**FIGURE 6:**
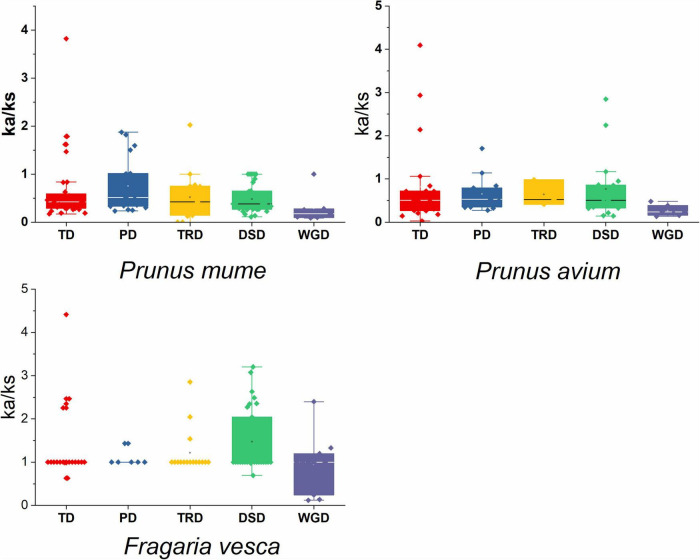
Ka/Ks values of GAox gene family in three Rosaceae species. Comparison of Ka/Ks values for different modes of gene duplications. WGD, whole-genome duplication; PD, proximal duplication; TRD, transposed duplication; TD, tandem duplication; DSD, dispersed duplication.

In sweet cherry, apricot, and strawberry, the average Ks ratio of DSD generated duplicated pairs was 1.87, 1.24, and 0.69, respectively (Supplementary Table 4). In *P. mume*, *P. avium*, and *F. vesca*, the Ka/Ks value of duplicated pairs was 1, indicating that GAox genes were subjected to intense purifying selection. However, as demonstrated in Supplementary Table 4 and Figure 6, sweet cherry, Japanese apricot, and strawberry duplicated gene pairs exhibit higher Ka/Ks values (> 1), suggesting that the GAox gene increment has a complex evolutionary history.

### GO Annotation Enrichment Analysis

**FIGURE 7:**
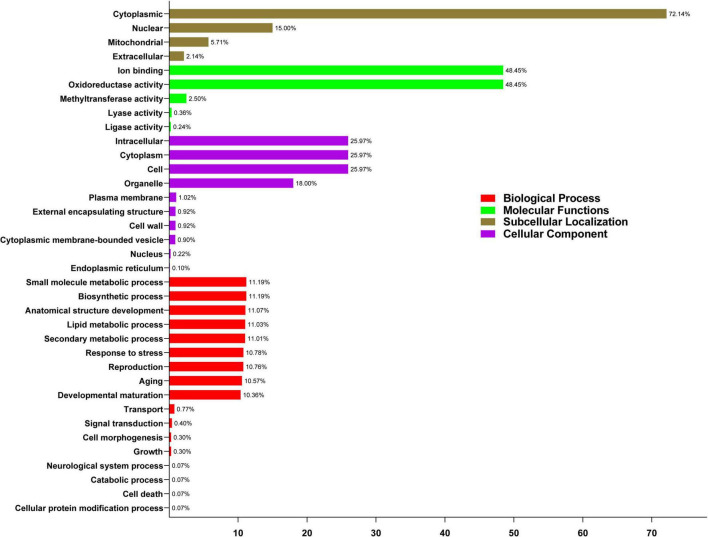
Gene ontology (GO) annotation of PvGAox proteins. The GO annotation was achieved based on three categories, biological process (BP), molecular function (MF), and cellular component (CC). The numbers on the abscissa show the number of predicted proteins.

The prediction of various functions, such as biological processes, molecular functions, subcellular localization, and cellular components, was analyzed. In addition, under the four primary groups, 140 GAox proteins were divided into 36 functional groups based on protein similarity. According to the ontology of biological processes, the highest percentage was engaged in small-molecule metabolic and biosynthetic processes (11.19%), while GAox genes involved 11.07% in anatomical structure development. Among the GAox genes, 11.01, 11.03%, 10.78%, 10.76%, 10.57%, and 10.36% of genes were found to be involved in secondary metabolic processes, lipid metabolic processes, response to stress, reproduction, aging, and developmental maturation, respectively. Moreover, some GAox genes were involved in transport (0.77%), signal transduction (0.40%), cell morphogenesis (0.30%), growth (0.30%), and cell death (0.07%). In the ontology of cellular components, GAox genes with the highest percentage (25.97%) are involved in cells, intracellular, and cytoplasm, followed by the organelle (18%), plasma membrane (1.02%), external encapsulating structure (.92%), cell wall (0.92%), cytoplasmic membrane-bound vesicle (0.92%), nucleus (0.22%), and endoplasmic reticulum (0.10%). Furthermore, molecular functions ontology revealed that ion binding and oxidoreductase activity are identical and have a high proportion (48.45%) when compared to methyltransferase activity (2.50%), lyase activity (0.36%), and ligase activity (0.24%) (Supplementary Table 5). CELLOGO tool software was used to estimate subcellular localization. These findings suggest that the majority of GAox genes (72.14%) are found in the cytoplasm, with the remaining genes engaged in nuclear (15%), mitochondrial (5.71%), and extracellular activities (2.14 %) (Figure 7).

### Promotor Analysis

**FIGURE 8:**
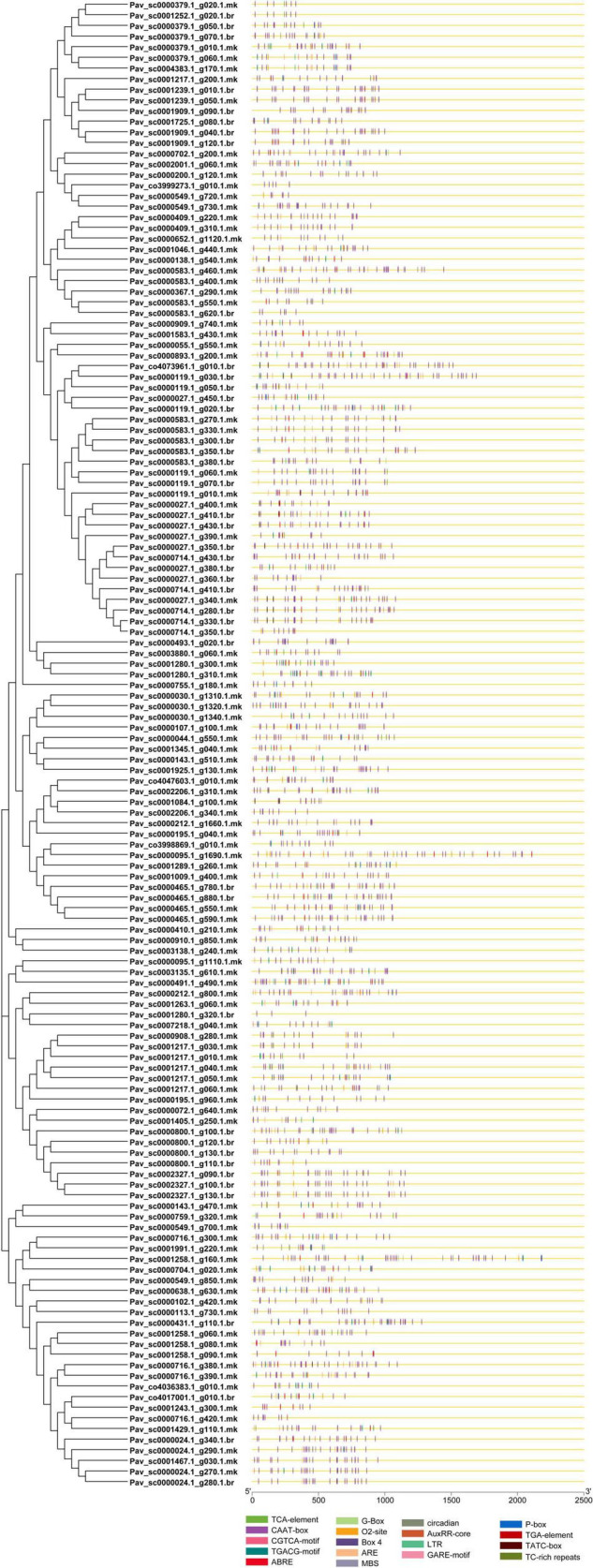
Predicted *cis*-elements in the promoter regions of the *PavGAox* genes. All promoter sequences (2 kb) were analyzed. The *PavGAox* genes are shown on the left side of the figure. The scale bar at the bottom indicates the length of promoter sequence.

The binding specificity of the TFs is determined by the *cis*-regulatory element in the promoter region and plays a key role in transcription regulation. *PavGAox cis*-regulatory elements were identified to be involved in phytohormone (gibberellin, ABA, auxin, and salicylic acid response elements) and stress responses (low temperature, light, and drought). Numerous *cis*-regulatory elements were found to be involved in the hormonal response, such as auxin (TGA element, AuxRR-core) response element, gibberellin response element (GARE-motif, P-box, TATC-box), MeJA (TGACG, CGTCA-motif, TGACG-motif), and ABA (ABRE). Simultaneously, stress–response elements associated with light responsiveness (G-Box, Box 4), low-temperature reactivity (LTR), defense and stress responsiveness (TC-rich repeats), zein metabolism regulation (O2-site), anaerobic induction (ARE), circadian control (circadian), and the MYB binding site (MBS) involved in drought induction were recognized (Figure 8). The CAAT-Box and ABRE motifs were discovered in most *PavGAox* genes, and their numbers were higher in the promoters of *Pav sc0001258.1 g160.1.mk*, *Pav sc0000583.1 g350.1.br*, *Pav sc0000893.1 g200.1.mk*, and *Pav sc0000143.1 g470.1.mk* than in *PavGAox* genes (Figure 8). This suggested that the ABRE and CAAT-Box motifs play a significant role in the stress response. These *cis*-elements had a role in ABA responsiveness, as well as promoter and enhancer regions. Anaerobic induction response elements were found in 4% of *PavGAox* members, whereas the MYB-binding site (MBS) implicated in drought induction was found in 3% of total members. Moreover, light responsiveness of *cis*-acting regulatory elements (G-Box, Box 4) comprised only 2% of the total *PavGAox* members. The phytohormone response-associated *cis*-elements, including TGACG motif (3%), P-Box (1%), TCA-element (1%), and TGA-element (1%), were also revealed, which are related to MeJA, gibberellin, salicylic acid, and auxin responses, respectively (Supplementary Table 6 and Supplementary Figure 1). Furthermore, we identified GAox *cis*-elements essential to plant growth/development, comprising 2% of members having 02-site that are associated with zein metabolic responsiveness.

### Expression Profile of *PavGAox* Genes in Different Dormancy Stages

**FIGURE 9:**
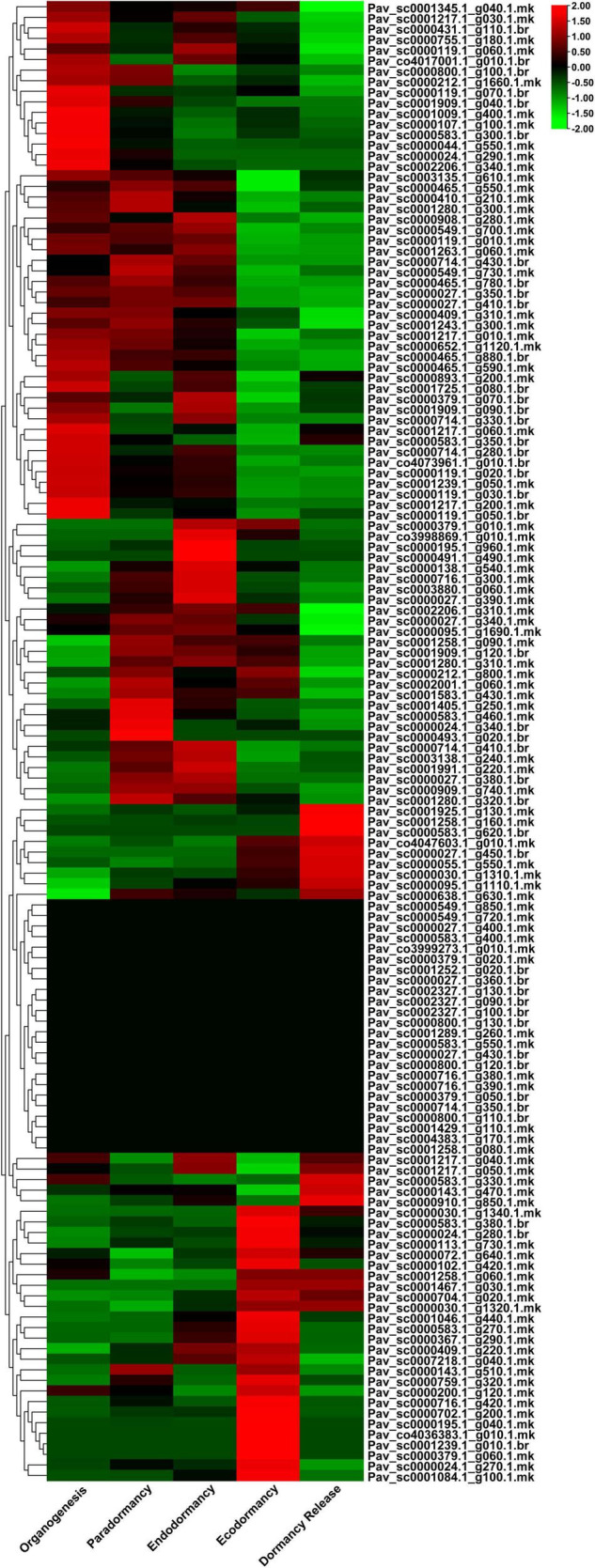
Transcriptomic analysis of 140 *PavGaox* genes in different dormancy-related phase data (organogenesis, paradormancy, endodormancy, ecodormancy, and dormancy release). Red, black, and green represent high, low, and no expression levels, respectively.

We examined the expression profile of *PavGAox* genes in sweet cherry at various dormancy phases using RNA-seq data (Figure 9) to authenticate the expression patterns of GAox family members in dormancy. RNA-seq data from earlier research were performed in five phases of dormancy (organogenesis, paradormancy, ecodormancy, and dormancy release). The spatial and temporal expression profiles of GAox members in various phases of dormancy in sweet cherry were investigated using transcriptomic data. The gene expression pattern was analyzed using transcripts per kilobase million (TPM) measurements. All *PavGAox* members were classified into three groups based on the expression behavior in different dormancy phases (Supplementary Table 7). In the first category, some members did not show any expression in any stage of dormancy, such as *Pav_sc0001289.1_g260.1.mk* and *Pav_sc0000800.1_g120.1.br*; 24 members (17.14%) remained silent in all phases. In the second category, 38 GAox family members (27.14%) were included which remain silent some specific phases and expressed their peak expression in some crucial stages of dormancy like *Pav_sc0000195.1_g040.1.mk*, *Pav_sc0000379.1_g060.1.mk*, and *Pav_sc0001239.1_g010.1.br* exhibited their peak expression only in ecodormancy phase, while they remained silent in all other dormancy stages. This phenomenon revealed that GAox family members had some stage-specific expression behaviors. In the third category, 78 members (55.71%) were included. All these *PavGAox* members were upregulated in all phases of dormancy, and some members indicated some specific expression pattern like *Pav_sc0000465.1_g880.1.br*, *Pav_sc0000027.1_g350.1.br*, *Pav_sc0000409.1_g310.1.mk*, *Pav_sc0001009.1_g400.1.mk*, and *Pav_sc0001909.1_g040.1.br* showed peak expression in the initial stage, but as the dormancy phase proceed, the expression pattern peak decreased (Figure 9).

### Expression of Candidate PavGAox Genes in Different Organs Along With Abiotic Stress by qRT-PCR

**FIGURE 10:**
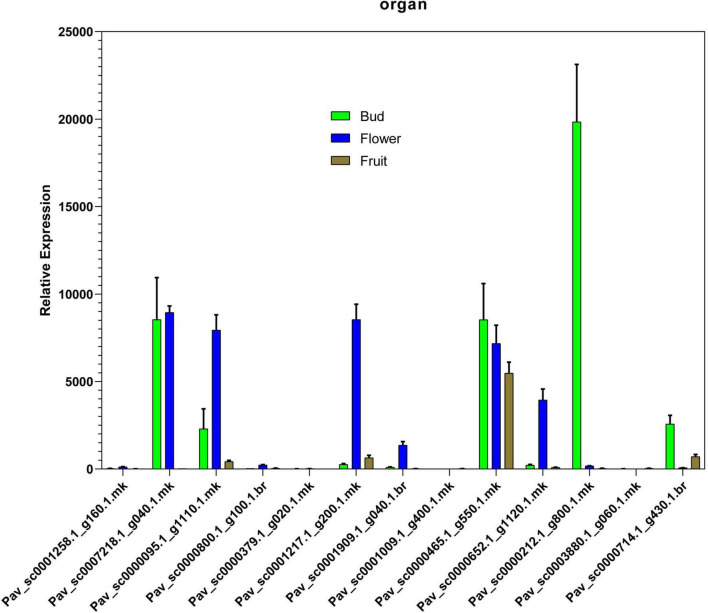
Relative expression patterns of *PavGAox* genes through qRT-PCR on different tissues (bud, flower, and fruit). Mean ± SE of three biological replicates (each having three technical replicates).

**FIGURE 11:**
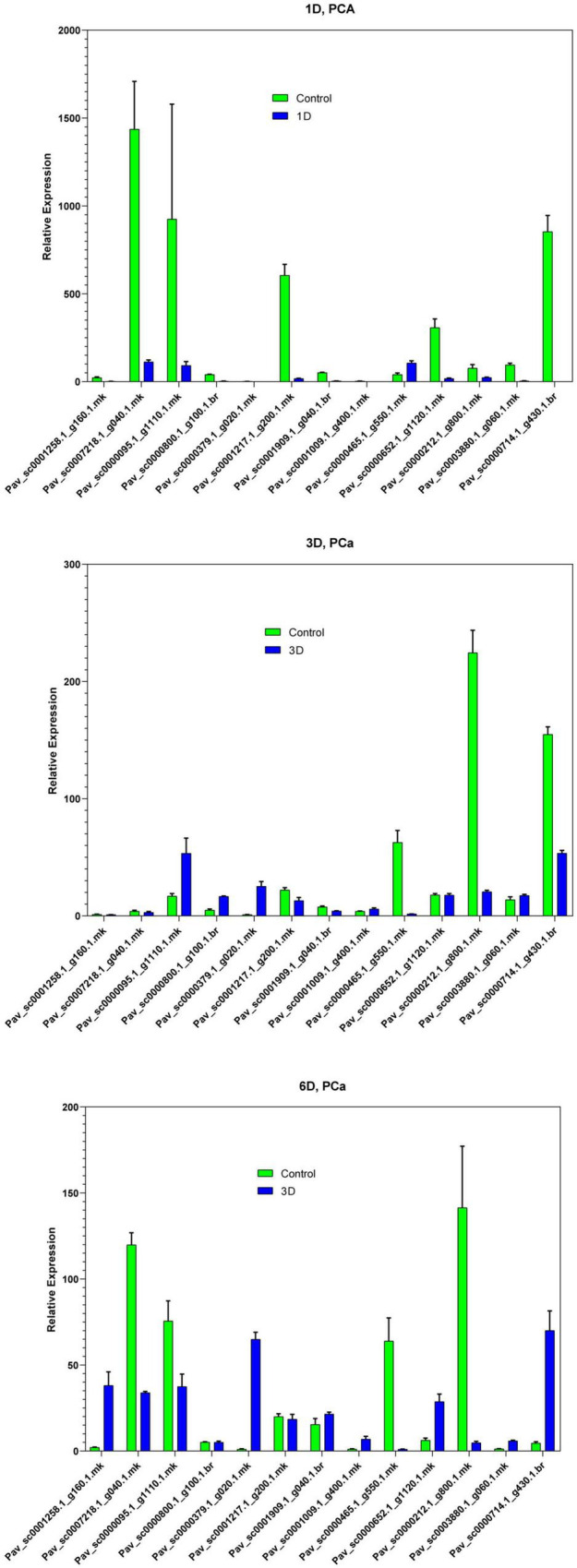
Expression profiles of selected *PavGaox* genes on the bud before (control) or 1D, 3D, and 6D after treatment with prohexadione calcium. Mean ± SE of three biological replicates (each having three technical replicates).

**FIGURE 12:**
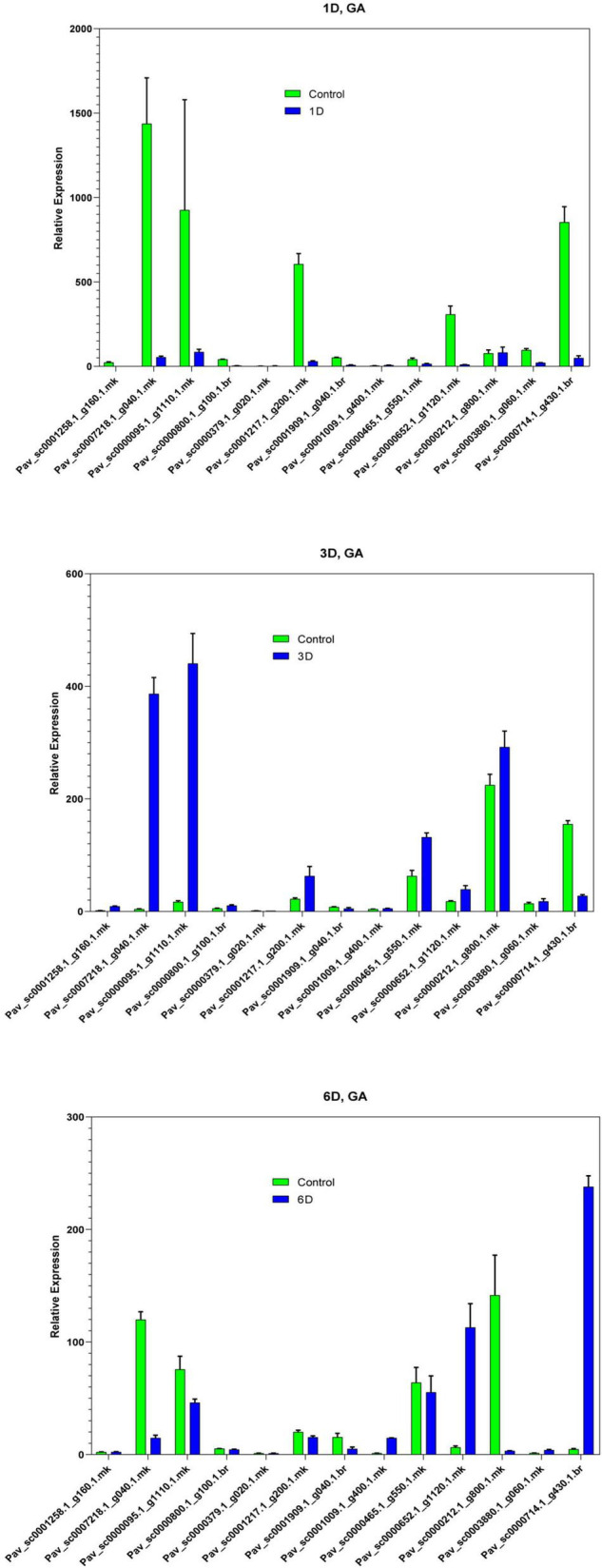
Expression profiles of selected *PavGaox* genes on the bud before (control) or 1D, 3D, and 6D after treatment with GA. Mean ± SE of three biological replicates (each having three technical replicates).

In the current investigation, we also analyzed the selected GAox member’s expression in sweet cherry parts like buds, flowers, and fruits through qRT-PCR. Several GAox genes had an increased expression at the flower development stage like *Pav_sc0001258.1_g160.1.mk*, *Pav_ sc0000095.1_g1110.1.mk*, *Pav_sc0000652.1_g1120.1.mk*, *Pav_ sc0000800.1_g100.1.br*, *Pav_ sc0000379.1_g020.1.mk*, *Pav_sc00 01909.1_g040.1.br*, *Pav_sc0007218.1_g040.1.mk*, and *Pav_sc00 01217.1_g200.1.mk*, while some genes like *Pav_sc0000 465.1_g550.1.mk*, *Pav_sc0000212.1_g800.1.mk*, and *Pav_sc000 0714.1_g430.1.br* were upregulated and expressed their peak expression in bud development. Few *PavGAox* members were involved in fruit maturation like *Pav_sc0003880.1_g060.1.mk*. These findings illustrated that GAox members are highly active in development stages, and they played a key role in plant initial development stages (Figure 10). Totally, 13 gibberellin-dioxygenases were validated by qRT-PCR analysis at 1D (day), 3D (days), and 6D (dayd) following GA treatment and PCa treatment to identify the important GA stress-responsive candidates in *Prunus avium*. These results illustrated that most of the genes were upregulated and expressed their peak expression at 3D when treated with GA_4+7_ like *Pav_sc0000095.1_g1110.1.mk*, *Pav_sc0007218.1_g040.1.mk*, *Pav_sc0000212.1_g800.1.mk*, and *Pav_sc0000465.1_g550.1.mk.* These members remained silent at 1D but revealed their peak expression at 3D. One *PavGAox* member (*Pav_sc0000714.1_g430.1.br*) also expressed its peak expression at 6D (Figure 11). However, some members like *Pav_sc0000379.1_g020.1.mk*, *Pav_sc0001258.1_g160.1.mk*, *Pav_sc0000714.1_g430.1.br*, and *Pav_sc0000652.1_g1120.1.mk* were downregulated with the treatment of GA, but these members expressed their peak expression at 6D when treated with anti-GA treatment (PCa) (Figures 11, 12). These findings lead us to understand their key role in maintaining the GA biosynthesis.

### Subcellular Localization

**FIGURE 13:**
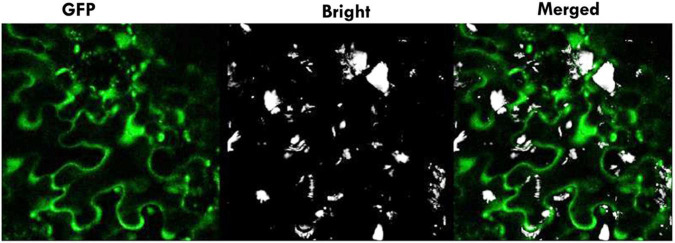
Subcellular localization of *Pav_sc0000465.1_g550.1.mk* in tobacco epidermal cells. Transient expression of *Pav_sc0000465.1_g550.1.mk* was investigated in epidermal cells of tobacco using a confocal microscope. For the subcellular localization of p35S:: *Pav_sc0000465.1_g550.1.mk*: eGFP, recombinants were detected by *Agrobacterium*-mediated infiltration.

To analyze the subcellular localization of GAox protein, the constructed plasmid p35S:: *Pav_sc0000465.1_g550.1.mk* 1::eGFP binary construct was transiently introduced into the lower epidermis of tobacco (*Nicotiana benthamiana*) leaves by the Agrobacterium-mediated infiltration method (Figure 13). The results demonstrate that the green fluorescence protein (GFP) fused with *Pav_sc0000465.1_g550.1.mk* was spread throughout the entire cellular structures, including the nucleus and plasma membrane (Figure 13), which is consistent with the previous findings (Zhang et al., 2019). These findings concluded that *PavGAox* indeed localized in the plasma membrane and nucleus.

## Discussion

Dormancy is an important trait that permits temperate woody perennials to endure harsh winter environments. Plants go into dormancy when their growth is restricted, and they are metabolic signals that cause it: paradormancy, endodormancy, and ecodormancy (Lang et al., 1987; Horvath et al., 2003). GAs have a vital role in regulating diverse activities like dormancy, flower induction, another development, and elongation all through the life cycle of plants. Gibberellin is the most significant phytohormone in the regulation of dormancy (Finch-Savage and Leubner-Metzger, 2006). The regulation of GA production determines the amount of bioactive GAs in plant tissues (Yamaguchi, 2008). GA biosynthesis is predominantly controlled by GA 20-oxidase (GA20ox) and GA 3-oxidase (GA3ox) processes. However, GA inactivation is mostly controlled by GA 2-oxidase (GA2ox). Genes encoding these enzymes have been found in a variety of crop species, including barley (*Hordeum vulgare*), wheat (*Triticum aestivum*), and rice (*Oryza sativa*) (Spielmeyer et al., 2004; Yamaguchi, 2008), and their expression regulates GA levels for dormancy (Spielmeyer et al., 2004; Yamaguchi, 2008). Gibberellin-dioxygenase genes have been linked to several important diversified processes (Hernández-García et al., 2021). In Arabidopsis, rice, and other plants, comprehensive and integrative investigations of gibberellin-dioxygenase genes have been published (Huang et al., 2015).

Our present study is a rigorous and extensive whole-genome evolutionary investigation of GAox members based on three Rosaceae genomes. A total number of 140 GAox genes were discovered from *Prunus avium* (sweet cherry), 146 from *Prunus mume* (Japanese apricot), and 113 from *F. vesca* (strawberry). Based on phylogenetic analysis, GAox genes from the three Rosaceae species (*F. vesca*, *P. avium*, and *P. mume*) were grouped into six distinct subfamilies (A, B, C, D, E, and F) (Figure 1). The consequence of gene gain or loss might have happened all throughout the evolutionary change. Functional divergence resulted from the addition and elimination of certain GAox gene members. Moreover, our investigation revealed that all gibberellin-dioxygenase genes had at least one domain. In *P. avium*, a maximum number of domains (4) were observed in *Pav_sc0001258.1_g160.1.mk*, while *Pav_sc0001263.1_g060.1.mk* had three domains. In *P. mume*, *Pm013434*, *Pm020609*, and *Pm020619* contained the highest number (3) of domains, while in *F. vesca*, a maximum number (4) of domains were observed in *FvH4_2g03200.1*. Supplementary Table 2 provides the full details of Rosacea species (physiochemical characterization) of the 399 GAox-protein. Additionally, the length of amino acids in GAox proteins varied significantly, ranging from 101 to 1,451 kDa. Furthermore, previous finding revealed that the differences across clades might be due to distinct roles, exon/intron variability, and motif structure. Moreover, gene structure analysis (intron–exon) was analyzed and was found to play an important in the evolution of various genes (Mustafin and Khusnutdinova, 2015). The 399 GAox genes investigated in the present study have different number of introns and exons, demonstrating that the GAox genes of the three Rosaceae species are highly diverse. The GAox genes have introns/exons ranging from 1/1 (*Pav sc0000716.1 g300.1.mk*, *Pm004966*, and *FvH4 2g27140.1*) to 29/30 (*Pm004369*) (Figure 1). The evolutionary study revealed that multigene families have originated mostly as a result of structural genetic variation (Xu et al., 2012). In addition, most of the genes in the same subfamily had comparable motif compositions. The GAox members were functionally varied, as shown by the organization and abundance of the 20 different inter- and intra-species motif categories (Figure 2). The conserved motif examination of the GAox gene family revealed the evolutionary history and classification of the sweet cherry GAox genes. GAox genes show much diversity in introns/exons and motif structure, implying high complexity in sweet cherry. Similarities of motifs and exon/intron structure and composition supported the evolutionary contribution of GAox members and varied their activities in sweet cherry within the same and distinct subfamilies. According to collinearity and phylogeny relationship, the sweet cherry (*P. avium*) genome and the other four Rosaceae genomes have stronger conserved region, which revealed a possible evolutionary mechanism between them. Gene duplication is an important process in all plants for developing genetic diversity, which might help organisms in adapting to climatic change (Alvarez-Buylla et al., 2000; Katiyar et al., 2012). Gene duplication events (TD, PD, WGD, DSS, and TRD) of the GAox family were revealed in three Rosaceae species (*P. avium*, *Fragaria vesca*, and *P. mume*) to help researchers better understand gene evolution and novel functions. Five types of duplications (dispersed duplication (DSD), tandem duplication (TD), proximal duplication (PD), whole-genome duplication (WGD), and (TRD) transposed duplication) were investigated in the GAox gene family, all of which contribute to the proliferation of certain genes in plants in diverse ways (Si et al., 2019). Tandem and whole-genome duplications played a vital role in gene family expansion like in *Vitis vinifera* (grape) (Guo et al., 2014). AP2/ERF and WRKY genes were highly diversified due to TD and WGD, while duplication events played a key role for MYB gene family evolution in *Setaria italic* (Muthamilarasan et al., 2014). Current findings suggested that DSDs and TDs are important in the proliferation of GAox genes in Rosaceae species. WGDs may play a role in the GAox gene family expansion (Supplementary Table 4). These findings revealed that duplications are essential in GAox gene expansion, particularly DSD and TDs occurring frequently in Rosaceae species. Ka/Ks values are used to calculate the selection pressure on genes, as well as their evolutionary history (Cao et al., 2013; Manzoor et al., 2020, 2021a). In general, the ka/ks value less than 1 indicated purifying selection, a ka/ks ratio higher than 1 indicated positive selection, and ka/ks value of 1 indicated neutral selection (Starr et al., 2003; Manzoor et al., 2021a). Positive selection is defined as a value larger than 1, while purifying selection is denoted as a ratio less than 1. Neutral selection is characterized as a value of 1 (Zhang et al., 2014; Manzoor et al., 2020). As per the findings of all ka/ks ratios of paralogous genes, purifying selection might be predominantly responsible for GAox protein functions. Gene expression analysis could provide the most valuable insights for elucidating diverse types of gene activities (Neilson et al., 2017). GAox genes have been found to have functional diversity in a variety of plants, including *Camellia sinensis* (Pan et al., 2017), *Vitis vinifera* (He et al., 2019), *Oryza sativa* (Liu et al., 2018), and *Zea mays* (Ci et al., 2021). In the current investigation, according to the phylogenetic tree, 13 *PavGAox* candidate genes were selected randomly from (subfamily A-F) for qRT-PCR in the sweet cherry buds, flowers, and fruits. Several GAox genes had an increased expression at the flower development like *Pav_sc0001258.1_g160.1.mk* and *Pav_sc0001217.1_g200.1.mk*, while some genes like *Pav_sc0000465.1_g550.1.mk* were upregulated and expressed their peak expression in bud development. Few *PavGAox* members were involved in fruit maturation like *Pav_sc0003880.1_g060.1.mk*. Current findings revealed that GAox members were highly active in the development stages, and they played a key role in the initial development stages in plants. However, transcriptional factors (TFs) exploit specific binding of *cis*-regulatory regions in target gene promoters to control them both regionally and functionally (Qiu, 2003). GAox genes include growth promoter and stress-related elements, such as CAAT-box, ABRE, LTR, and MBS (Figure 8 and Supplementary Table 6). Previous studies demonstrated that GAox genes have been engaged in growth and development, stress, hormones, and circadian rhythm regulation (He et al., 2019). GA regulation is also influenced by GAox genes (Pan et al., 2017). Some genes exclude gibberellin-related regions, suggesting that other components in the GA signaling pathway might be regulating GA levels in plants through influencing endogenous GA content fluctuations. The same phenomenon was also observed in grapes (He et al., 2019). Furthermore, numerous plants improve their abiotic/biotic stress response with hormone treatments like SA, MeJA, and ABA (Takanashi et al., 2014; Cheng et al., 2019; Manzoor et al., 2020). However, tissue specificity expression is a vital phenomenon of GAox members, which was also reported previously in tea plants (Nagar and Kumar, 2000) and rice (Huang et al., 2010). We also investigated the abiotic stress expressional behavior of selected candidate genes with GA_4+7_ and PCa. Maximum genes expressed their peak expression when treated with GA_4+7_, while some genes expressed themselves in downregulating patterns like *Pav_sc0001909.1_g040.1.br*, but the same gene upregulated when treated with PCa. The results revealed that all genes which exhibited their upregulated expression under GA treatment, remained silent after the treatment of PCa except *Pav_sc0000095.1_g1110.1.mk*. The *PavGAox* member expressed its upregulation in both treatments at 3 days. These findings revealed that this member had a crucial role in balancing the GA and ABA hormones, which are key hormones for maintaining all the dormancy processes. Furthermore, some *PavGAox* members, such as *Pav_sc0000800.1_g100.1.br* and *Pav_sc0001009.1_g400.1.mk*, did not present the significant expression and remained silent in both treatments, as well as in all dormancy phases too (RNA-Seq). This phenomenon illustrated that these members did have not some role in the dormancy mechanism, but they had some other special functions. These all findings revealed that GAox family regulates the diverse process in the plants (Figures 11, 12).

The RNA-seq data were also analyzed in different stages of bud dormancy. The results illustrated that GAox genes had highly stage-specific expression pattern. Some members like *Pav_co4017001.1_g010.1.br*, *Pav_sc0000024.1_g340.1.br*, and *Pav_sc0000024.1_g270.1.mk* expressed the same expression pattern. These members revealed their expression only in all dormancy phases, except the dormancy release phase. Current results authenticated that these members only took part in the dormancy process, and they remained silent as the dormancy was released. Moreover, the results also revealed that the GAox genes followed the same tissue-specific expression ability. Members of GAox had a vital role in dormancy initiation in sweet cherry. Some members, including *Pav_sc0000044.1_g550.1.mk* and *Pav_sc0000107.1_g100.1.mk*, had the highest expression as compared to all other members, which demonstrated that these genes are crucial for dormancy initiation. Previous research also illustrated that GAox had stage-specific expression pattern, such as in grape and tea plants (Pan et al., 2017; He et al., 2019). As we summarized, 66.42% members upregulated in organogenesis, while in paradormancy, endodormancy, and ecodormancy upregulation patterns were 75.71, 74.28, and 72.85%, respectively. Only 66% of members indicated expression in dormancy release. There was a wide range of tissue expression patterns, indicating that gibberellin-dioxygenase genes have a wider functional range, which would lead to diverse plant morphogenesis. The same expression profile of gibberellin dioxygenase gene functional diversity was also confirmed in maize (Ci et al., 2021). In a nutshell, our research contributes to a better understanding of the GAox family’s functional divergence in sweet cherries and suggests that positive selection may have played a vital role in the evolution of GAox genes. However, the concise function and mechanism of these *PavGAox* genes must be investigated further.

## Conclusion

In the current investigation, we identified 399 GAox members based on the publicly available Rosaceae genome (*F. vesca*, *P. mume*, and *P. avium*), which were further classified into six subfamilies (A-F). Phylogeny analysis, gene structure (intron/exon), conserved motif, *cis*-regulatory elements, and GO annotation analyses illustrated that GAox members in *P. avium* are conserved and divergently associated through other species. Also the bioinformatics analysis were performed, including collinearity relationship, physicochemical characterization, chromosomal position, conserved domain, ka/ks, and transcriptomic analysis. The expansion of GAox genes might be aided by dispersed duplication (DSD) and whole-genome duplication (WGD). Additionally, the functional variety of GAox genes, which would promote to various morphogeneses in plant development, were shown by the diversity of tissue expression patterns through transcriptome data and qRT-PCR. Furthermore, some genes, such as *Pav co4017001.1 g010.1.br*, *Pav sc0000024.1 g340.1.br*, and *Pav sc0000024.1 g270.1.mk*, were identified as key genes for regulating GAs that play important roles in plant development. This comprehensive analysis of gibberellin-dioxygenase genes in sweet cherry will be the foundation for future investigation of the genetic improvement and functional features.

## Data Availability Statement

All data generated or analyzed during this study are openly available the sweet cherry (*P. avium*), Japanese apricot (*P. mume*) sequences were downloaded from GDR (Genome Database for Rosaceae) (https://www.rosaceae.org). While strawberry (*Fragaria vesca*) was downloaded from Joint Genome Institute (JGI) Data Portal (http://www.jgi.doe.gov/). The *Arabidopsis thaliana* GAox protein sequences were obtained from TAIR website (https://www.arabidopsis.org/). RNA-seq data of sweet cherry was downloaded from the NCBI website (https://www.ncbi.nlm.nih.gov/sra) of different fruit developmental stages with accession number SRR8984402, SRR8984360, SRR8984367, SRR8984382, SRR8984344, SRR8984381, SRR8984359, SRR8984403, SRR8984342, and SRR8984366 of *P. avium* on various dormancy phases (organogenesis, paradormancy, endodormancy, and ecodormancy).

## Author Contributions

IAS and MM conceived and designed the experiments and wrote the manuscript. IAS, MM, IHS, FA, XL, AS, SF, SJ, JW, and MA contributed to reagents, materials, and analysis tools. CZ provided guidance on the whole manuscript. All authors read and approved the final manuscript.

## Conflict of Interest

The authors declare that the research was conducted in the absence of any commercial or financial relationships that could be construed as a potential conflict of interest.

## Publisher’s Note

All claims expressed in this article are solely those of the authors and do not necessarily represent those of their affiliated organizations, or those of the publisher, the editors and the reviewers. Any product that may be evaluated in this article, or claim that may be made by its manufacturer, is not guaranteed or endorsed by the publisher.

## Footnotes

1
https://www.rosaceae.org

2
http://www.jgi.doe.gov/

3
http://www.ebi.ac.uk/interpro/sea

4
http://smart.emblheidelberg.de

5
http://pfam.xfam.org

6
https://web.expasy.org/protparam

7
http://cello.life.nctu.edu.tw/cello2go/

8
http://itol.embl.de

9
http://gsds.gao-lab.org/

10
https://meme-suite.org/meme/

11
https://mapinspect.software.informer.com/

12
http://hmmer.org/

13
https://www.ncbi.nlm.nih.gov/Structure/lexington/lexington.cgi

14
https://bioinformatics.psb.ugent.be/webtools/plantcare/html/

15
https://popgenie.org/node/42

16
http://services.cbu.uib.no/tools/kaks

17
https://www.genscript.com/tools/pcr-primers-designer

## Supplementary Material

Supplementary Figure 1 Pie chart percentage of *cis* element in sweet cherry (*Prunus avium* L.).

Supplementary Figure 2 Chromosomal localization in sweet cherry (*Prunus avium* L.).

Supplementary Table 1 List of Primers used in qRT-PCR analysis.

Supplementary Table 2 Molecular characteristics of GAox genes in *Prunus mume*.

Supplementary Table 3 List of GAox orthologous gene pairs identified in *Malus domestica*, *Fragaria vesca Prunus mume* and *Prunus persica*.

Supplementary Table 4 Gene duplication events and synonymous and non-synonymous value of GAox gene family in *Fragaria vesca*.

Supplementary Table 5 Molecular process, biological process, Cellular components, and subcellular localization of GAox gene family in *Prunus avium*.

Supplementary Table 6 *Cis*-acting elements of GAox gene family in *Prunus avium*.

Supplementary Table 7 RNA-Seq data of Sweet Cherry on different bud dormancy stages.
