# Supplementary figures and images for: Evolutionary and Integrative Analysis of Gibberellin-Dioxygenase Gene Family and Their Expression Profile in Three Rosaceae Genomes (F. vesca, P. mume, and P. avium) Under Phytohormone Stress
Source: Front Plant Sci. 2022 Jul 7;13:942969. doi: 10.3389/fpls.2022.942969 (PMC9302438; doi:10.3389/fpls.2022.942969)

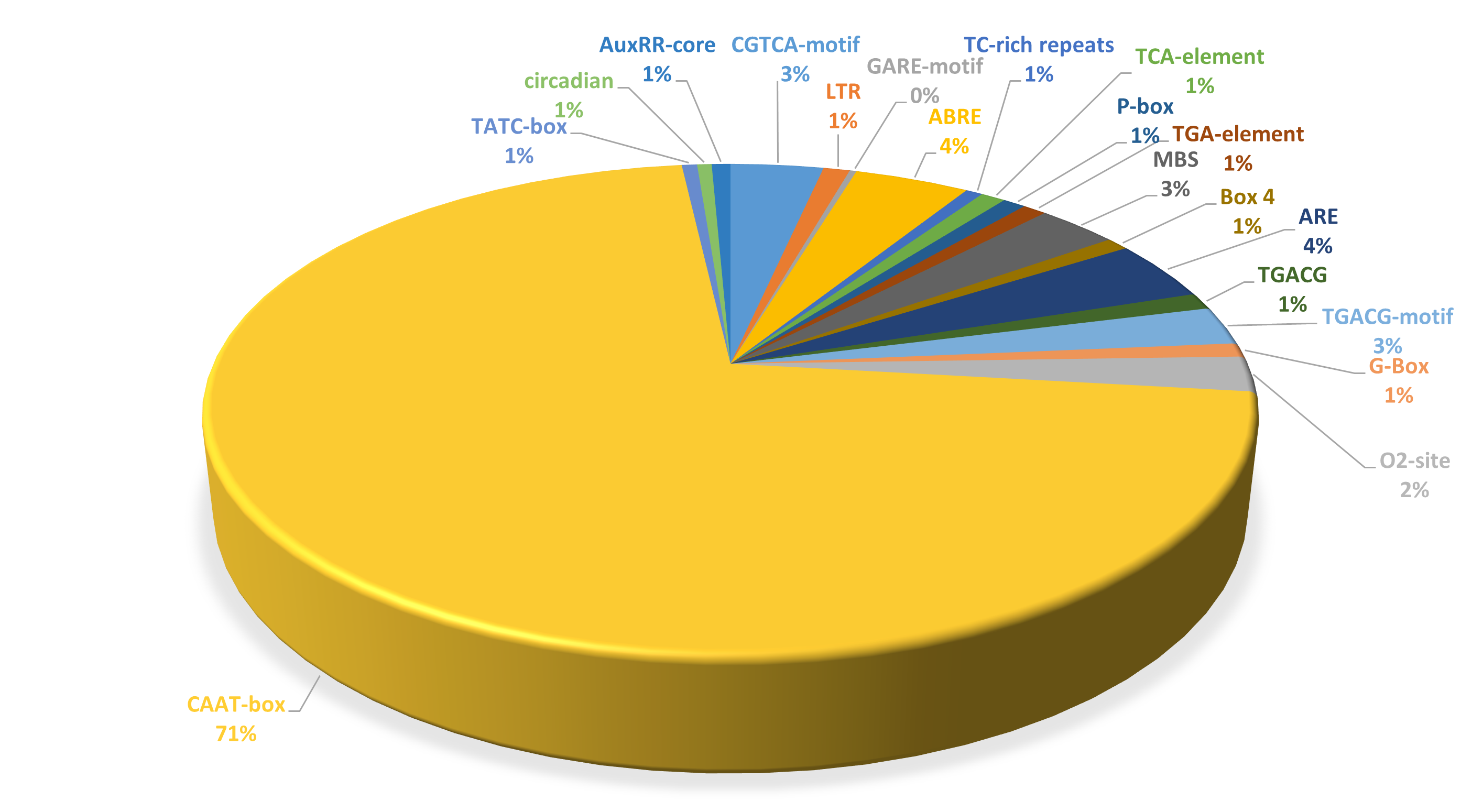

Supplement: Supplementary Figure 1 — Pie chart percentage of cis element in sweet cherry (Prunus avium L.). [file Data_Sheet_1.zip › Image 1.PNG]

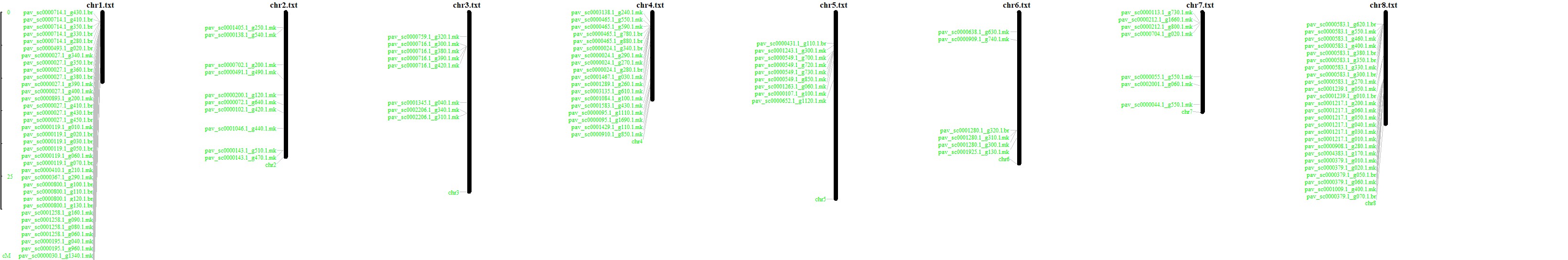

Supplement: Supplementary Figure 1 — Pie chart percentage of cis element in sweet cherry (Prunus avium L.). [file Data_Sheet_1.zip › Image 2.JPEG]
